# Seven new species of the *Exocelina ekari* group from New Guinea central and coastal mountains (Coleoptera, Dytiscidae, Copelatinae)

**DOI:** 10.3897/zookeys.1026.61554

**Published:** 2021-03-25

**Authors:** Helena Shaverdo, Suriani Surbakti, Bob Sumoked, Michael Balke

**Affiliations:** 1 Naturhistorisches Museum Wien, Burgring 7, 1010 Vienna, Austria Naturhistorisches Museum Wien Vienna Austria; 2 Department of Biology, Universitas Cendrawasih, Waena, Papua, Indonesia Universitas Cendrawasih Waena Indonesia; 3 Walian 2, Tomohon Selatan, N Sulawesi 95439, Indonesia Unaffiliated Tomohon Selatan Indonesia; 4 SNSB-Zoologische Staatssammlung München, Münchhausenstraße 21, D-81247 Munich, Germany SNSB-Zoologische Staatssammlung München Munich Germany; 5 GeoBioCenter, Ludwig-Maximilians-University, Munich, Germany GeoBioCenter Munich Germany

**Keywords:** Australasia, distribution, *
Exocelina
*, new species, systematics

## Abstract

Seven new species of the genus *Exocelina* Broun, 1886 are described from three different mountain ranges of New Guinea: *E. foja***sp. nov.**, *E. riberai***sp. nov.**, *E. apistefti***sp. nov.**, and *E. waaf***sp. nov.** from the Foja Mountains; *E. hudsoni***sp. nov.** from the Cyclops Mountains; *E. ekpliktiki***sp. nov.** and *E. oraia***sp. nov.** from Wano Land. All of them are placed into the *E. ekari* group based on the structure of their male genitalia. The species are characteristic dytiscid elements of the fauna of northern cost and the western part of central orogen of New Guinea. Two taxonomic notes are presented: *Exocelina athesphati* is a correct name for the recently described *Exocelina athesphatos* Shaverdo et al., 2020; *Exocelina bacchus* Balke, **nom. nov.** is a replacement name for *Exocelina bacchusi* (Balke, 1998), formerly Copelatus (Papuadytes) bacchusi Balke, 1998, a junior homonym of *Copelatus bacchusi* Wewalka, 1981.

## Introduction

Even after more than 20 years of research on New Guinea *Exocelina* diving beetles, the island’s rugged mountain regions continue to reveal new species (Shaverdo et al. 2020a, b). Our examination of specimens found in the northern Foja and Cyclops Mountains, as well as in the Wano Land, a mountain area of the western central orogen, revealed seven new species. So far, only *E. bewaniensis*Shaverdo et al., 2014 (*E. ekari* group) had been known from the Foja Mountains, where four additional new species were found. From the Cyclops Mountains, only *E. cyclops*Shaverdo et al., 2018 (*E. casuarina* group) was known to date; here we describe one additional new species from this steep mountain range. From the Wano Land, six closely related species had been described, which constitute a complex close to the *E. ekari* group (Shaverdo et al. 2017), as well as *E. sumokedi* Shaverdo & Balke, 2018 from the *E. casuarina* group. We studied unidentified material from this region and discovered the presence of *E. bewaniensis* and two new species.

All seven new species were found to belong to the *E. ekari* group. To date, this, the largest *Exocelina* species group, contains 63 species; 152 *Exocelina* species are now described from New Guinea and 209 species worldwide.

## Materials and methods

The material studied is housed in the following collections:

**KSP** Koleksi Serangga Papua, at the Biology Department of Universitas Cenderawasih (UNCEN), Wamena, Papua, Indonesia;

**MZB**Museum Zoologicum Bogoriense, Cibinong, Indonesia.

Our methods follow those described in detail in our previous articles (Shaverdo et al. 2012, 2014; Shaverdo and Balke 2014). The terminology to denote the orientation of the genitalia follows Miller and Nilsson (2003). All specimen data are quoted as they appear on the labels attached to the specimens. Label text is cited using quotation marks; comments in square brackets are ours. The following abbreviations were used: **MW** (maximum body width), **TL** (total body length), **TL-H** (total body length without head).

## Results

### Descriptions of the species from the Foja Mountains

#### Exocelina foja

Taxon classificationAnimaliaColeopteraDytiscidae

Shaverdo, Surbakti & Balke sp. nov.

B471A368-2E38-576C-8299-8670D6AE89EA

http://zoobank.org464A09DF-E5D6-4D1D-9455-59676F42C60D/

Figures 1 , 5 , 15

Exocelina nr. pseudosoppi #7286: Toussaint et al. 2021: figs 3–6.

##### Type locality.

Indonesia: Papua Province, Sarmi Regency, Foja Mts, 02°34'18.6"S, 138°43'02.1"E, 1700 m a.s.l.

##### Type material.

***Holotype*:** male “Indonesia: Papua, Foja Mountains, bog camp, 1700m, 23.v.-3.vi.2016, -2.571839 138.717250, Sumoked (Pap058)” (MZB).

***Paratypes*:** 26 males, 19 females with the same label as the holotype, three males with additional handwritten labels “creek A”, “creek C” and “creek D” (MZB, KSP). 1 male, 6 females “Indonesia (1700A): Papua, Foja Mountains, bog camp, 1700m, 23.v.-3.vi.2016, -2.571839 138.717250, Sumoked (Pap058)” (KSP). 4 males, 5 females “Indonesia (1700B): Papua, Foja Mountains, bog camp, 1700m, 23.v.-3.vi.2016, -2.571839 138.717250, Sumoked (Pap058)” (MZB, KSP). 2 males, 3 females “Indonesia (1700D): Papua, Foja Mountains, bog camp, 1700m, 23.v.-3.vi.2016, -2.571839 138.717250, Sumoked (Pap058)” (KSP). 13 males, 10 females “Indonesia: Papua, Foja Mountains, bog camp, 1700m, 23.v.-3.vi.2016,”, “-2.571839 138.717250, Sumoked (Pap058)”, two females with additional green text labels “7357” and “7358” (MZB, KSP). 2 males, 4 females “Indonesia: Papua, Foja Mountains, river camp, 1600m, 23.v.-3.vi.2016, -2.561006 138.711487, Sumoked (Pap059)”, one male with an additional handwritten label “forest near bog camp”, the other male with an additional green text label “7286” (MZB, KSP).

##### Description.

**Figures 1–4.**
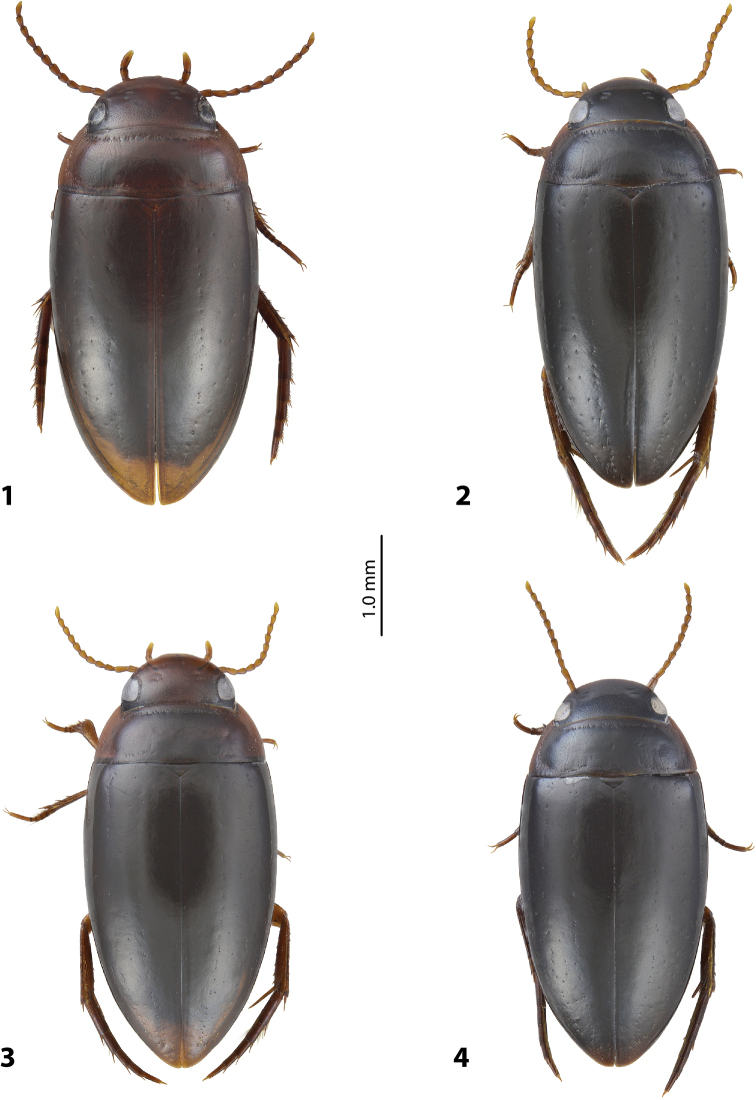
Habitus and colouration of holotype **1***Exocelina foja* sp. nov. **2***E. waaf* sp. nov. **3***E. riberai* sp. nov. **4***E. apistefti* sp. nov.

***Body size and form*:** Beetle small: TL-H 3.30–3.85 mm, TL 3.70–4.30 mm, MW 1.80–2.10 mm (holotype: TL-H 3.85 mm, TL 4.30 mm, MW 2.10 mm), with oblong-oval habitus (Fig. 1).

***Colouration*:** Dorsally dark brown to piceous, with paler head and sides of pronotum (Fig. 1). Head more or less uniformly dark brown to reddish brown, darker around eyes, or slightly paler anteriorly; pronotum dark brown to piceous on disc and distinctly paler (to yellowish red) anteriorly, posteriorly, and especially laterally; dark area on disc sometimes represented just as median band; elytra dark brown to piceous, with reddish brown sutural lines; head appendages and legs yellowish red to reddish brown. Teneral specimens paler.

***Surface sculpture*:** Shiny dorsally, with fine punctation and microreticulation. Head with dense and coarse punctation (spaces between punctures 0–3 times size of punctures), distinctly finer and sparser anteriorly and posteriorly; diameter of punctures equal to diameter of cells of microreticulation. Pronotum with distinctly finer and sparser punctation than on head. Elytra with very sparse and fine punctation, almost invisible. Pronotum and elytra with weakly impressed microreticulation; head with stronger microreticulation. Metaventrite and metacoxa distinctly but weakly microreticulate, metacoxal plates with longitudinal strioles and transverse wrinkles. Abdominal ventrites with weak microreticulation, strioles, and fine sparse punctation, coarser and denser on two terminal abdominal ventrites.

***Structures*:** Pronotum with narrow lateral bead. Base of prosternum and neck of prosternal process with ridge, slightly rounded anteriorly. Blade of prosternal process lanceolate, relatively narrow, slightly convex medially, with distinct bead and few setae. Abdominal ventrite 6 broadly rounded apically.

**Figures 5, 6.**
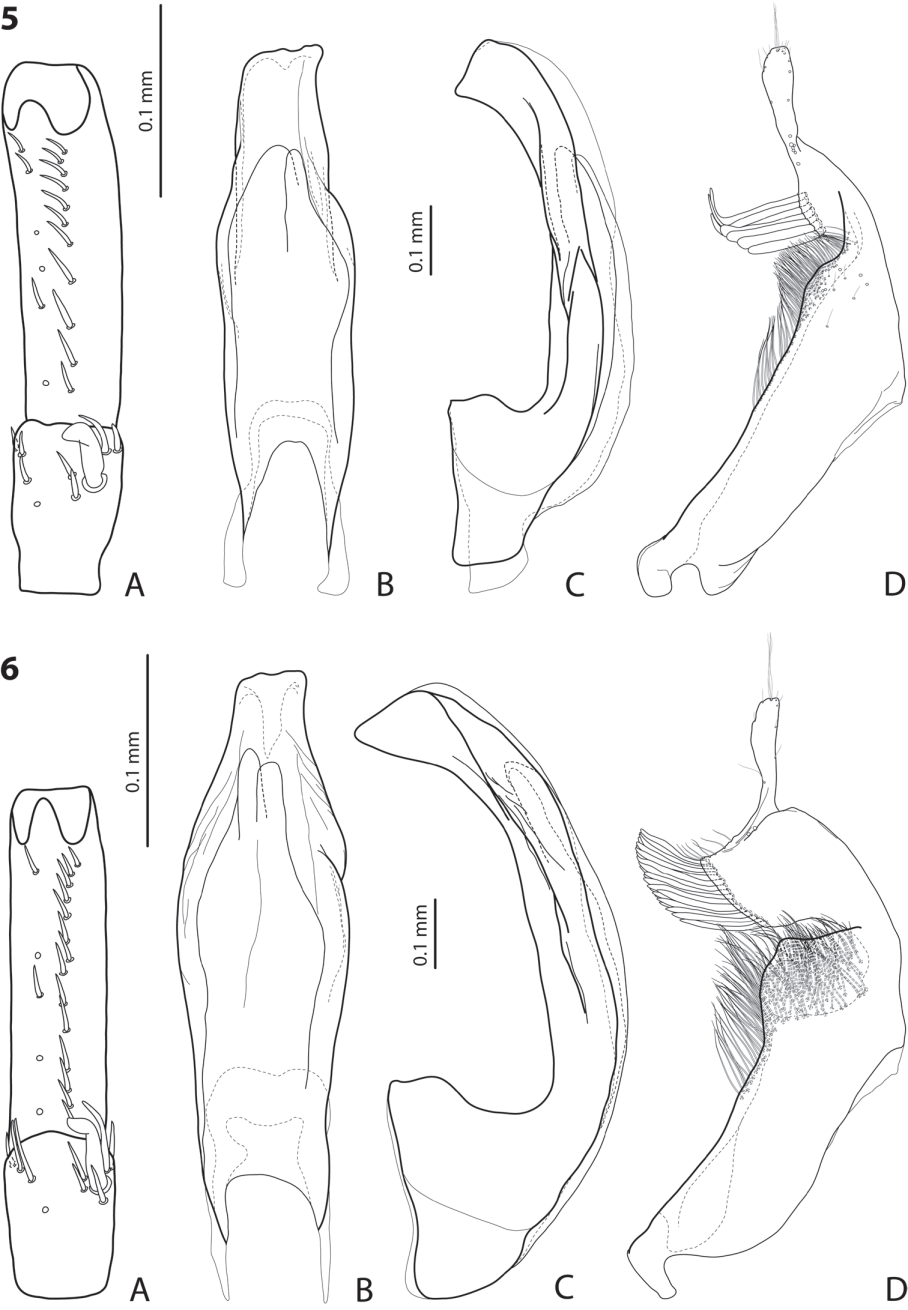
**5***Exocelina foja* sp. nov., holotype **6***E. waaf* sp. nov., holotype **A** right protarsomeres 4 and 5 in ventral view **B** median lobe in ventral view **C** median lobe in lateral view **D** right paramere in external view.

***Male*:** Antenna simple. Pro- and mesotarsomeres 1–3 not dilated, narrow. Protarsomere 4 cylindrical, narrow, with medium-sized, thick, strongly curved anterolateral hook-like seta. Protarsomere 5 ventrally with anterior row of eleven and posterior row of six short setae (Fig. 5A). Median lobe with distinctly discontinuous outline; in lateral view, almost straight, with apex broad, curved downwards, and sharply pointed at tip; in ventral view, with distinct submedian constriction, distal part narrower than proximal one, apex truncate (Fig. 5B, C). Paramere with strong notch on dorsal side and subdistal part relatively large and elongate; subdistal setae very few, dense and flattened: three upper longer, thinner, curved at apex and four lower shorter, almost straight, thicker; proximal setae hair-like, numerous, dense, but distinctly more inconspicuous than subdistal ones (Fig. 5D). Abdominal ventrite 6 broadly rounded, with 5–9 lateral striae on each side.

***Female*:** Pro- and mesotarsi not modified. Abdominal ventrite 6 without striae.

##### Affinities.

The species evidently belongs to the *E. ekari* group due to the discontinuous outline of its median lobe. Within the group, it can be placed close to *E. oceai*Shaverdo et al., 2012 due to presence of the pronotal lateral bead. Shape of its median lobe is similar to that of *E. pseudosoppi*Shaverdo et al., 2012; setation of the paramere is very characteristic.

##### Distribution.

Indonesia: Papua Province. This species is known only from Foja Mountains, from and near the type locality (Fig. 15).

##### Habitat.

The specimens were collected from small, shallow forest creeks.

##### Etymology.

The species is named after Foja Mountains. The name is a noun in the nominative singular standing in apposition.

#### Exocelina apistefti

Taxon classificationAnimaliaColeopteraDytiscidae

Shaverdo, Surbakti & Balke sp. nov.

7427E30D-F6B8-5974-B293-7820FFBC61A3

http://zoobank.org/7862C435-10FA-4ED3-B73E-BACCE3D10EBB

Figures 4 , 8 , 15

Exocelina nr. brahminensis #7287: Toussaint et al. 2021: figs 3–6.

##### Type locality.

Indonesia: Papua Province, Sarmi Regency, Foja Mts, 02°34'18.6"S, 138°43'02.1"E, 1700 m a.s.l.

##### Type material.

***Holotype*:** male “Indonesia: Papua, Foja Mountains, bog camp, 1700m, 23.v.-3.vi.2016, -2.571839 138.717250, Sumoked (Pap058)”, “7287” [green text] (MZB).

***Paratypes*:** 2 females with the same label as the holotype (KSP). 1 male, 1 female “Indonesia: Papua, Foja Mountains, river camp, 1600m, 23.v.-3.vi.2016, -2.561006 138.711487, Sumoked (Pap059)” (MZB, KSP).

##### Description.

***Body size and form*:** Beetle small: TL-H 3.45–3.7 mm, TL 3.8–4.05 mm, MW 1.85–2 mm (holotype: TL-H 3.5 mm, TL 3.9 mm, MW 1.9 mm), with oblong-oval habitus (Fig. 4).

***Colouration*:** Dorsally piceous, with paler lateral sides of pronotum (Fig. 4). Head piceous, with slightly paler, dark brown, anterior margin; pronotum piceous, slightly paler towards lateral sides, lateral sides brown to dark brown, yellowish to reddish brown at anterior angles; elytra uniformly piceous; head appendages and proximal part of legs yellowish brown, legs distally brown.

***Surface sculpture*:** Shiny dorsally, with fine microreticulation and almost invisible punctation on elytra. Head with dense and coarse punctation (spaces between punctures 0–3 times size of punctures), distinctly finer and sparser anteriorly and posteriorly; diameter of punctures equal to diameter of cells of microreticulation. Pronotum with distinctly finer and sparser punctation than on head. Elytra with very sparse and fine punctation, almost invisible. Elytra with weakly impressed microreticulation; pronotum and especially head with stronger microreticulation. Metaventrite and metacoxa distinctly but weakly microreticulate, metacoxal plates with longitudinal strioles and transverse wrinkles. Abdominal ventrites with weak microreticulation, strioles, and fine sparse punctation, coarser and denser on two last abdominal ventrites.

***Structures*:** Pronotum without lateral bead. Base of prosternum and neck of prosternal process with ridge, slightly rounded anteriorly. Blade of prosternal process lanceolate, relatively narrow, slightly convex medially, with distinct bead and few setae. Abdominal ventrite 6 broadly rounded apically.

***Male*:** Antenna simple. Pro- and mesotarsomeres 1–3 not dilated, narrow. Protarsomere 4 cylindrical, narrow, with medium-sized, thick, strongly curved anterolateral hook-like seta. Protarsomere 5 ventrally with anterior row of 13 and posterior row of six short setae (Fig. 8A). Median lobe with distinctly discontinuous outline; in lateral view, almost straight, with apex broad, curved downwards, and pointed at tip; in ventral view, with distinct submedian constriction, distal part narrower than proximal one, apex deeply and narrowly concave (Fig. 8B, C). Paramere with strong notch on dorsal side, with median notch tip sharply pointed, and subdistal part large and elongate; subdistal setae long and dense, of two kind: more numerous upper ones thin and lower setae shorter, thicker and flattened; proximal setae hair-like, distinctly more inconspicuous than subdistal ones (Fig. 8D). Abdominal ventrite 6 broadly rounded, with 9–11 lateral striae on each side.

**Figures 7, 8.**
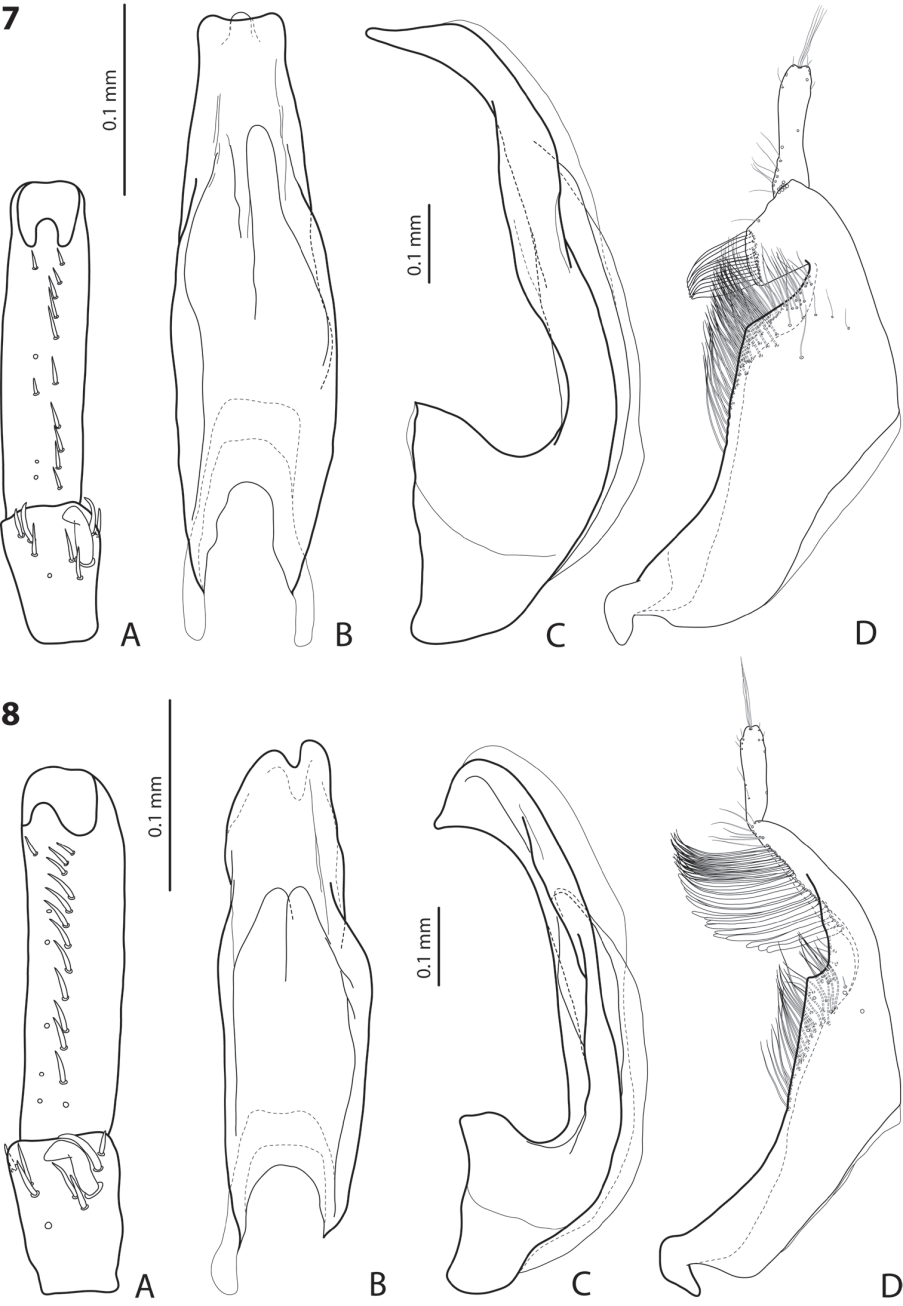
**7***Exocelina riberai* sp. nov., holotype **8***E. apistefti* sp. nov., holotype **A** right protarsomeres 4 and 5 in ventral view **B** median lobe in ventral view **C** median lobe in lateral view **D** right paramere in external view.

***Female*:** Pro- and mesotarsi not modified. Abdominal ventrite 6 without striae.

##### Affinities.

The species evidently belongs to the *E. ekari* group due to the discontinuous outline of its median lobe. The species is very similar to *E. brahminensis*Shaverdo et al., 2012 in general appearance and structure of male genitalia, especially in the sharply pointed median notch tip of paramere, but differs from it in lager size (TL-H 3.15–3.3 mm for *E. brahminensis*), darker dorsal colouration, shape of the median lobe, and paramere setation.

##### Distribution.

Indonesia: Papua Province. This species is known only from Foja Mountains, from and near the type locality (Fig. 15).

##### Habitat.

The specimens were collected from small, shallow forest creeks.

##### Etymology.

The species name *apistefti* derives from Greek *απίστευτος* (feminine *απίστευτη*) meaning unbelievable. The name is an adjective in the nominative singular.

#### Exocelina riberai

Taxon classificationAnimaliaColeopteraDytiscidae

Shaverdo, Surbakti & Balke sp. nov.

367E9AA5-9B90-5821-8898-68232A82AAEA

http://zoobank.org/44511064-4B35-4242-8018-898B0BAB4B0C

Figures 3 , 7 , 15

Exocelina “Foja” #7282: Toussaint et al. 2021: figs 3–6.

##### Type locality.

Indonesia: Papua Province, Sarmi Regency, Foja Mts, N Waaf Village, 02°22'29.6"S, 138°44'19.9"E, 115 m a.s.l.

##### Type material.

***Holotype*:** male “Indonesia: Papua, Foja Mountains N foot, N Waaf vill, 115m, 23.v.-3.vi.2016, -2.374874 138.738855, Sumoked (Pap060)” (MZB).

***Paratypes*:** 3 males, 5 females with the same label as the holotype (MZB, KSP). 2 males “Indonesia: Papua, Foja Mountains N foot, N Waaf vill, pondok, 150m, 4.–7.vi.2016, -2.406142 138.74399, Sumoked (Pap061)”, one male with an additional green text label “7282” (KSP).

##### Description.

***Body size and form*:** Beetle small to medium-sized: TL-H 3.45–3.85 mm, TL 3.8–4.3 mm, MW 1.85–2.1 mm (holotype: TL-H 3.6 mm, TL 4.1 mm, MW 1.9 mm), with oblong-oval habitus (Fig. 3).

***Colouration*:** Dorsally dark brown to piceous, usually with paler, reddish brown, head and pronotum (Fig. 3). Head reddish brown, reddish anteriorly, dark brown around eyes; pronotum reddish brown to brown, with darker, to dark brown, disc; elytra dark brown, sometimes with reddish brown sutural lines; head appendages yellow, legs yellowish red to reddish brown. One specimen (from locality Pap061) piceous, with dark brown pronotal lateral sides. Most specimens from locality Pap060 teneral, therefore, paler.

***Surface sculpture*:** Shiny dorsally, with inconspicuous, almost invisible elytral punctation and weakly impressed microreticulation. Head with relatively fine and sparse punctation (spaces between punctures 1–4 times size of punctures); diameter of punctures smaller than diameter of cells of microreticulation. Pronotum with finer, sparser, and more evenly distributed punctation than on head, often inconspicuous. Elytra with very sparse and fine punctation, almost invisible. Elytra with weakly impressed microreticulation; pronotum and especially head with stronger microreticulation. Metaventrite and metacoxa distinctly but weakly microreticulate, metacoxal plates with longitudinal strioles and very weak transverse wrinkles. Abdominal ventrites with weak microreticulation, strioles, and punctation visible only on two last abdominal ventrites.

***Structures*:** Pronotum without lateral bead, in some specimens (especially characteristic for females) with bead traces or even with narrow bead on lateral sides of pronotum. Base of prosternum and neck of prosternal process with distinct ridge, slightly rounded anteriorly. Blade of prosternal process lanceolate, relatively broad, slightly convex, with distinct lateral bead and few setae. Abdominal ventrite 6 broadly rounded.

***Male*:** Antenna simple. Pro- and mesotarsomeres 1–3 not dilated, narrow. Protarsomere 4 cylindrical, narrow, with medium-sized, thick, distinctly curved anterolateral hook-like seta. Protarsomere 5 ventrally with anterior row of ten and posterior row of five short setae (Fig. 7A). Median lobe with distinctly discontinuous outline; in lateral view, almost straight, with apex narrow, curved downwards, and strongly protruding at tip forming a long thin prolongation; in ventral view, with weak submedian constriction, distal part narrower than proximal one, apex slightly and evenly concave, with distinct protruding tip (Fig. 7B, C). Paramere with strong notch on dorsal side and subdistal part subquadrate, large and broad; subdistal setae dense, rather short, flattened; proximal setae hair-like, numerous, dense, and long (Fig. 7D). Abdominal ventrite 6 broadly rounded, with 13–16 lateral striae on each side.

***Female*:** Pro- and mesotarsi not modified. Abdominal ventrite 6 without lateral striae. Bead traces or even with narrow bead on lateral margins pronotum present in majority of females.

##### Affinities.

The new species evidently belongs to the *E. ekari* group due to the discontinuous outline of its median lobe. The species is similar to *E. pinocchio* Shaverdo & Balke, 2014 in general appearance and shape of median lobe, but differs from it in more straight apical prolongation of the median lobe and in subquadrate, large and broad subdistal part of the paramere (distinctly more elongate in *E. pinocchio*) and its setation.

##### Distribution.

Indonesia: Papua Province. This species is known only from Foja Mountains, from and near the type locality (Fig. 15).

##### Habitat.

The specimens were collected from shaded waterholes on a riverbank.

##### Etymology.

The species is named to honour Dr Ignacio Ribera Galán, a leading water beetle specialist and our dear colleague who passed away on 15 April 2020. The name is a noun in the genitive case.

#### Exocelina waaf

Taxon classificationAnimaliaColeopteraDytiscidae

Shaverdo, Surbakti & Balke sp. nov.

3F398A22-22BB-5096-8728-D1949338C2C4

http://zoobank.org/F172B13F-AF66-40E4-B0C3-67267C6811C3

Figures 2 , 6 , 15

Exocelina nr. utowaensis #7281: Toussaint et al. 2021: figs 3–6.

##### Type locality.

Indonesia: Papua Province, Sarmi Regency, Foja Mts, N Waaf Village, 02°22'29.6"S, 138°44'19.9"E, 115 m a.s.l.

##### Type material.

***Holotype*:** male “Indonesia: Papua, Foja Mountains N foot, N Waaf vill, 115m, 23.v.-3.vi.2016, -2.374874 138.738855, Sumoked (Pap060)” (MZB).

***Paratypes*:** 7 males, 2 females with the same label as the holotype, one male with an additional green text label “7281” (MZB, KSP).

##### Description.

***Body size and form*:** Beetle small to medium-sized: TL-H 3.55–3.75 mm, TL 3.9–4.2 mm, MW 1.95–2.0 mm (holotype: TL-H 3.65 mm, TL 4.1 mm, MW 1.95 mm), with oblong-oval habitus (Fig. 2).

***Colouration*:** Dorsally piceous, with paler lateral sides of pronotum (Fig. 2). Head piceous, with slightly paler, dark brown, anterior margin; pronotum piceous, slightly paler towards lateral sides, lateral sides brown to dark brown, yellowish red to reddish brown at anterior angles; elytra uniformly piceous or with dark brown sutural lines; head appendages and proximal part of legs yellowish brown, legs distally brown.

***Surface sculpture*:** Shiny dorsally, with inconspicuous, almost invisible elytral punctation and weakly impressed microreticulation. Head with uneven, sparse punctation (spaces between punctures 1–4 times size of punctures); diameter of punctures smaller than or almost equal to diameter of cells of microreticulation; punctation sparser and finer anteriorly and posteriorly. Pronotum with distinctly finer, sparser, and more evenly distributed punctation than on head. Elytra with very sparse and fine punctation, almost invisible. Elytra and pronotum with weakly impressed microreticulation; head with stronger microreticulation. Metaventrite and metacoxa distinctly but weakly microreticulate, metacoxal plates with longitudinal strioles and transverse wrinkles. Abdominal ventrites with weak microreticulation, strioles, and fine punctation.

***Structures*:** Pronotum without lateral bead. Base of prosternum and neck of prosternal process with distinct ridge, slightly rounded anteriorly. Blade of prosternal process lanceolate, narrow, convex, with distinct lateral bead and few setae. Abdominal ventrite 6 concave apically.

***Male*:** Antenna simple (Fig. 2). Pro- and mesotarsomeres 1–3 not dilated, relatively narrow. Protarsomere 4 cylindrical, narrow, with medium-sized, long, relatively slender, strongly curved anterolateral hook-like seta. Protarsomere 5 ventrally with anterior row of 14 and posterior row of five short setae (Fig. 6A). Median lobe with distinctly discontinuous outline; in lateral view, almost straight, with large, evenly tapering and curved downwards apex; in ventral view, with weak submedian constriction, distal part distinctly narrower than proximal one, apex truncate (Fig. 6B, C). Paramere with strong notch on dorsal side and subdistal part subquadrate, large and broad; subdistal setae long, dense, thick, and flattened; proximal setae hair-like, numerous, more inconspicuous than subdistal ones (Fig. 6D). Abdominal ventrite 6 distinctly concave, with 9–11 lateral striae on each side.

***Female*:** Pro- and mesotarsi not modified. Abdominal ventrite 6 slightly concave, without lateral striae.

##### Affinities.

The new species evidently belongs to the *E. ekari* group due to the discontinuous outline of its median lobe. The species is very similar to *E. utowaensis*Shaverdo et. al., 2012 in general appearance, apically concave abdominal ventrite 6, and structure of the male genitalia, but differs from in more slender male antennae and shape of the median lobe and paramere.

##### Distribution.

Indonesia: Papua Province. This species is known only from the type locality in Foja Mountains (Fig. 15).

##### Habitat.

The specimens were collected from shaded waterholes on a riverbank.

##### Etymology.

The species is named after Waaf Village. The name is a noun in the nominative singular standing in apposition.

### Key to the species from the Foja Mountains

Since five different species are now known from the Foja Mountains, it is worth providing a key to identify them. All species belong to the *E. ekari* group and are similar to each other in their external morphology. Therefore, the key is based mostly on characters of the male genitalia. Because of that, females cannot be often assigned to species and should be identified in association with males from the same population.

**Table:** 

1	Pronotum with narrow lateral bead. Median lobe and paramere as in Fig. 5	*** foja ***
–	Pronotum without lateral bead, sometimes (especially in females) with bead traces or even narrow bead, in this case, several specimens of population should be checked	**2**
2	Abdominal ventrite 6 concave apically. Median lobe and paramere as in Fig. 6	*** waaf ***
–	Abdominal ventrite 6 broadly rounded	**3**
3	Apex of median lobe very strongly protruding, forming long, thin prolongation in lateral view (Fig. 7C)	*** riberai ***
–	Apex of median lobe broad, short, and pointed at tip in lateral view	**4**
4	Apex of median lobe broader in lateral view; in ventral view, deeply and narrowly concave (Fig. 8B, C). Paramere with strong dorsal notch and notch tip sharply pointed. Subdistal part of paramere elongate, with upper, hair-like setae more numerous and strong (Fig. 8D)	*** apistefti ***
–	Apex of median lobe narrower in lateral view; in ventral view, shallowly and evenly concave (Figs 21–23C, D in Shaverdo et al. 2014). Paramere with weaker dorsal notch and notch tip absent. Subdistal part of paramere rounded, with upper, hair-like setae less numerous and weak (Figs 21–23E in Shaverdo et al. 2014)	*** bewaniensis ***

### Descriptions of the species from the Cyclops Mountains

Only two species are known from the Cyclops Mountains: *E. cyclops* Shaverdo & Balke, 2018 from the *E. casuarina* group and the newly described *E. hudsoni* sp. nov. from the *E. ekari* group. They can be easily distinguished due to smaller body size of *E. cyclops* (TL-H 3.0–3.25 mm), its reddish dorsal colouration, unmodified male antennae, and different structure of the male genitalia (Shaverdo et al. 2018).

#### Exocelina hudsoni

Taxon classificationAnimaliaColeopteraDytiscidae

Shaverdo, Surbakti & Balke sp. nov.

EA4BBD77-3A61-5559-8CA1-83393CFC82E5

http://zoobank.org/95FBA44C-42AA-4F8F-8919-244FD403A912

Figures 9 , 10 , 15

##### Type locality.

Indonesia: Papua Province, Jayapura Regency, Cyclops Mts, 1880 m a.s.l.

##### Type material.

***Holotype*:** male “Indonesia: Papua, Cyclops Mountains, below summit, 1880m, ii.201, Sentani Naturalist Club (Pap70)” (MZB).

***Paratypes*:** 7 females with the same label as the holotype (MZB, KSP).

##### Description.

**Figures 9, 10.**
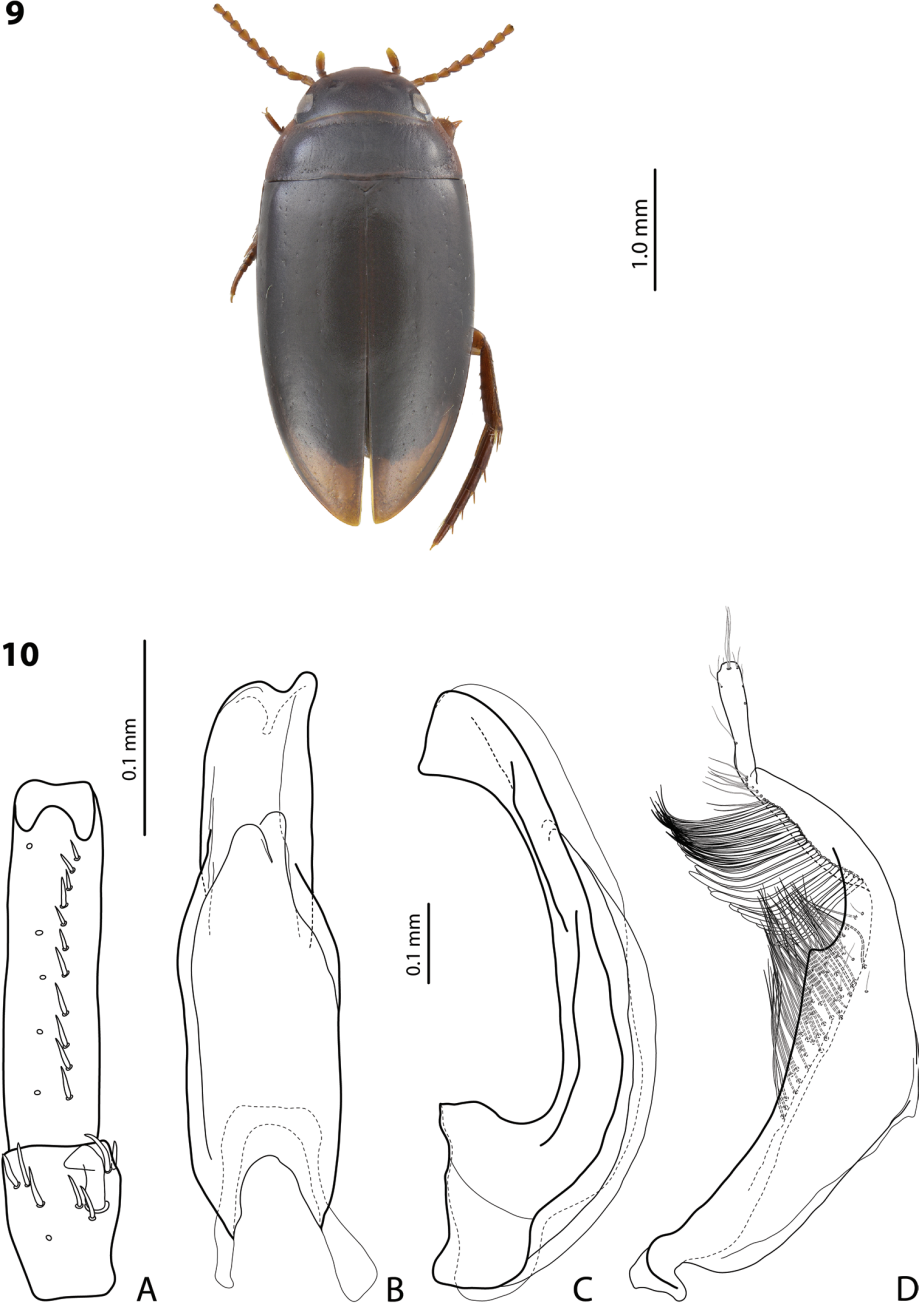
*Exocelina hudsoni* sp. nov., holotype **9** habitus and colouration **10** male structures **A** right protarsomeres 4 and 5 in ventral view **B** median lobe in ventral view **C** median lobe in lateral view **D** right paramere in external view.

***Body size and form*:** Beetle small to medium-sized: TL-H 3.4–3.75 mm, TL 3.75–4.2 mm, MW 1.8–2.05 mm (holotype: TL-H 3.4 mm, TL 3.8 mm, MW 1.8 mm), with oblong-oval habitus (Fig. 9).

***Colouration*:** Dorsally piceous, with paler head and pronotum (Fig. 9). Head piceous in posterior half and dark brown in anterior half; pronotum piceous on disc, sometimes narrowly, and brown to dark brown on sides, yellowish red to reddish brown at anterior angles; elytra piceous, with brown to dark brown sutural lines; head appendages and proximal part of legs yellowish brown, legs distally reddish brown.

***Surface sculpture*:** Submatt dorsally, with inconspicuous elytral punctation and strongly impressed dorsal microreticulation. Head with sparse central punctation (spaces between punctures 1–4 times size of punctures), denser towards eyes; diameter of punctures smaller than diameter of cells of microreticulation; punctation relatively shallow. Pronotum with distinctly finer, sparser, and more evenly distributed punctation than on head. Elytra with very sparse and fine punctation, almost invisible. Dorsal surface with strongly impressed microreticulation, microreticulation weaker on elytra and stronger on pronotum and head. Metaventrite and metacoxa distinctly but weakly microreticulate, metacoxal plates with longitudinal strioles and very weak transverse wrinkles. Abdominal ventrites with weak microreticulation, strioles, and almost invisible punctation.

***Structures*:** Pronotum with lateral bead. Base of prosternum and neck of prosternal process with distinct ridge, slightly rounded anteriorly. Blade of prosternal process lanceolate, relatively broad, convex, with distinct lateral bead and few setae. Abdominal ventrite 6 broadly rounded.

***Male*:** Antennomeres 4–10 slightly but distinctly enlarged (Fig. 9). Pro- and mesotarsomeres 1–3 not dilated, narrow. Protarsomere 4 cylindrical, narrow, with large, thick, strongly curved anterolateral hook-like seta. Protarsomere 5 ventrally with anterior row of eleven and posterior row of five short setae (Fig. 10A). Median lobe with distinctly discontinuous outline; in lateral view, almost straight, with apex broad, curved downwards, and pointed at tip; in ventral view, with distinct submedian constriction, distal part narrower than proximal one, apex asymmetrical, narrowly concave (Fig. 10B, C). Paramere with strong notch on dorsal side, with median notch tip sharply pointed, and subdistal part large and elongate; subdistal setae long, dense, curved at apex, and of two different types: upper setae thinner, more hair-like and lower setae thick and flattened; proximal setae hair-like, more inconspicuous than subdistal ones (Fig. 10D). Abdominal ventrite 6 broadly rounded, with nine lateral striae on each side.

***Female*:** Antennomeres 4–10 stout. Pro- and mesotarsi not modified. Abdominal ventrite 6 without lateral striae.

##### Affinities.

The species evidently belongs to the *E. ekari* group due to the discontinuous outline of its median lobe. The species is very similar to *E. brahminensis* and *E. apistefti* sp. nov. in general structure of male genitalia, especially in the sharply pointed median notch tip of paramere, but differs from them in submatt dorsal surface due to stronger microreticulation, presence of pronotal bead, enlarged antennomeres 4–10, shape of the median lobe, and setation of the paramere.

##### Distribution.

Indonesia: Papua Province. This species is known only from the type locality in Cyclops Mountains (Fig. 15).

##### Habitat.

The specimens were collected from small puddles at low spot of a small ravine.

##### Etymology.

This species is named after Hudson Wild, a most dedicated naturalist and community worker in Papua. The name is a noun in the genitive case.

### Descriptions of the species from the Wano Land

Ten species are now recorded from the Wano Land: *E. sumokedi* of the *E. casuarina* group, six species, mentioned in the Introduction, from the complex close to the *E. ekari* group (Shaverdo et al. 2017: 109, key), two species described herein, and the newly recorded *E. bewaniensis*. Diagnostic characters of latter three are discussed below; since they belong to the *E. ekari* group, they could be easily distinguished from the other *Exocelina* species occurring in this region (see Shaverdo et al. 2017, 2018).

#### Exocelina ekpliktiki

Taxon classificationAnimaliaColeopteraDytiscidae

Shaverdo, Surbakti & Balke sp. nov.

A5C44E67-EC75-5DD8-A53C-6E2F4D41083B

http://zoobank.org/5869A521-DFF6-45B6-9481-96A9C0D9F3D2

**Figures 11, 12.**
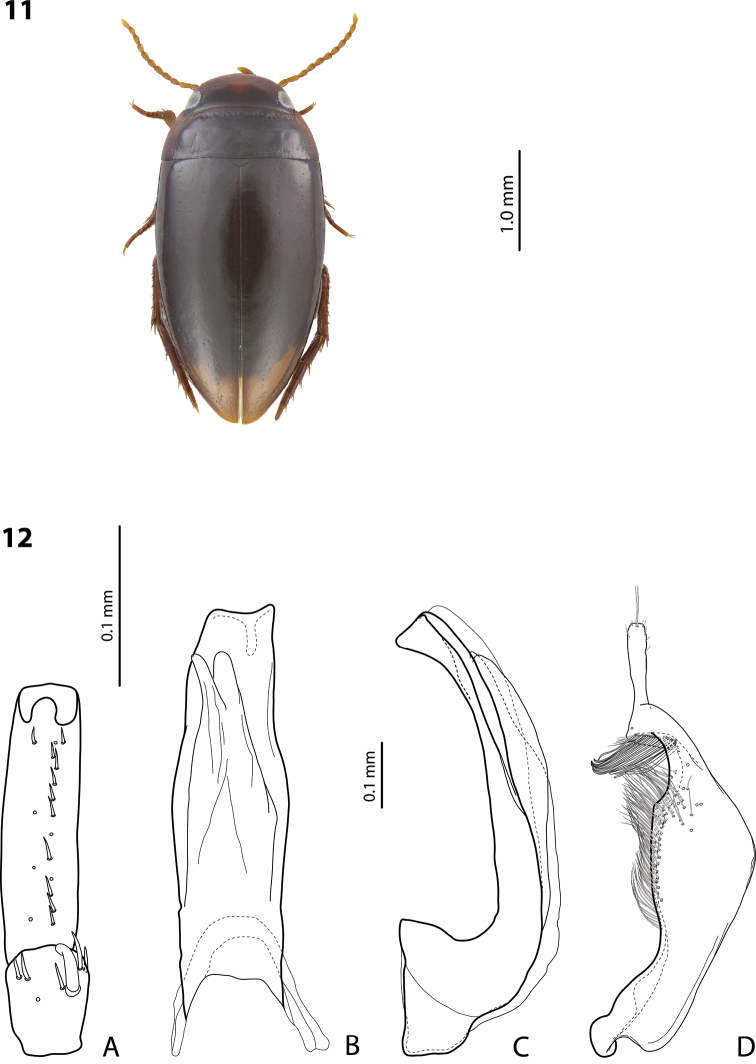
*Exocelina ekpliktiki* sp. nov., holotype **11** habitus and colouration **12** male structures **A** right protarsomeres 4 and 5 in ventral view **B** median lobe in ventral view **C** median lobe in lateral view **D** right paramere in external view.

Figures 11 , 12 , 15

Exocelina nr. oceai #6504: Toussaint et al. 2021: figs 3–6.

##### Type locality.

Indonesia: Papua Province, Puncak Regency, south from Iratoi, 03°54'20.4"S, 137°12'03.2"E, 378 m a.s.l.

##### Type material.

***Holotype*:** male “Indonesia: Papua, S Iratoi, forest, 378m, 22.v.2015, -3,3904028 137,32009999, Pele & Sumoked (Pap037)” (MZB).

***Paratypes*: Puncak Regency**: 16 males, 8 females with the same label as the holotype (MZB, KSP). 3 males “Indonesia: Papua, S Iratoi, forest, 378m, 22.v.2015, -3,3904028031975 137,320099985226, Pele & Sumoked (Pap037)”, one male an additional label “KSP6983” [green text] (KSP). 5 males, 2 females “Indonesia: Papua, S Iratoi, forest, 553m, 22.v.2015, -3,3919226937 137,3235277, Pele & Sumoked (Pap038)” (KSP). 1 male, 1 female “Indonesia: Papua, S Iratoi, forest, 553m, 22.v.2015, -3,39192269369959 137,323527764528, Pele & Sumoked (Pap038)”, with additional green text labels “6989” and “6988”, respectively (KSP). 17 males, 11 females “Indonesia: Papua, S Iratoi, forest, 450m, 23.v.2015, near -3,39192 137,323527764528, Pele & Sumoked (Pap039)” (MZB, KSP). 2 males “Indonesia: Papua, S Iratoi, forest, 450m, 23.v.2015, near -3,391922694 137,323527764528, Pele & Sumoked (Pap039)”, with additional green text labels “6986” and “6987” (KSP). 13 males, 5 females “Indonesia: Papua, Wano Land, red clay creek nr cave, 1100m, 3.ix.2014, nr -3.587955 137.5114945, Bennji (Pap024)”, one male an additional label “6517” [green text] (MZB, KSP).

**Puncak Jaya Regency.** 3 males “Indonesia: Papua, S Iratoi, forest, 220m, 21.v.2015, -3,38095162063837 137,311441982164, Pele & Sumoked (Pap036)”, one male an additional label “6982” [green text] (MZB, KSP). 6 males, 6 females “Indonesia: Papua, Rouaffer, Iratoi, hill in forest, 164m, 6.ix.2014, -3,2403086 137,3329744, Pele & Sumoked (Pap028)”, two males with additional green text labels “6504” and “6505” (KSP).

##### Description.

***Body size and form*:** Beetle small: TL-H 2.95–3.35 mm, TL 3.35–3.65 mm, MW 1.6–1.8 mm, excluding the locality Pap024, (holotype: TL-H 3.15 mm, TL 3.55 mm, MW 1.7 mm), with oblong-oval habitus (Fig. 11), some specimens, especially teneral slightly egg-shaped.

***Colouration*:** Dorsally dark brown to piceous, with paler, reddish brown anterior half of head and lateral sides of pronotum (Fig. 11). Head dark brown posteriorly and largely (to half) reddish brown anteriorly; pronotum dark brown to piceous, with reddish to reddish brown lateral sides, sometimes also narrowly anteriorly and posteriorly leaving dark brown disc; elytra dark brown to piceous, with reddish brown sutural lines; head appendages yellow, legs yellowish red to reddish brown. Teneral specimens paler, to pale brown, with yellow anterior half of head and pronotal sides.

***Surface sculpture*:** Shiny dorsally, with inconspicuous, almost invisible elytral punctation and weakly impressed microreticulation. Head with relatively sparse punctation (spaces between punctures 1–3 times size of punctures), evidently finer and sparser anteriorly and posteriorly; diameter of larger punctures almost equal to diameter of cells of microreticulation. Pronotum with finer, sparser, and more evenly distributed punctation than on head, often inconspicuous. Elytra with very sparse and fine punctation, almost invisible. Pronotum and elytra with weakly impressed microreticulation, sometimes stronger on pronotal sides; head with microreticulation much stronger. Metaventrite and metacoxa distinctly microreticulate, metacoxal plates with longitudinal strioles and transverse wrinkles. Abdominal ventrites with distinct microreticulation, strioles, and very fine and sparse punctation.

***Structures*:** Pronotum with distinct but narrow lateral bead, in some specimens reduced at posterior angles. Base of prosternum and neck of prosternal process with distinct ridge, slightly rounded anteriorly. Blade of prosternal process lanceolate, relatively narrow, slightly convex, with distinct lateral bead and few setae. Abdominal ventrite 6 broadly rounded.

***Male*:** Antenna simple. Pro- and mesotarsomeres 1–3 narrow. Protarsomere 4 narrow, with medium-sized, slightly curved anterolateral hook-like seta. Protarsomere 5 ventrally with anterior row of 13 and posterior row of four short, pointed setae (Fig. 12A). Median lobe with distinctly discontinuous outline; in lateral view, almost straight, with curved downwards, broadly pointed apex; in ventral view, with weak submedian constriction, distal part only slightly narrower than proximal one, and evenly, shallowly concave apex (Fig. 12B, C). Paramere with strong notch on dorsal side and subdistal part short and small; subdistal setae long, dense, curved at apex, few lower ones slightly flattened; proximal setae numerous, dense, but weaker than subdistal ones (Fig. 12D). Abdominal ventrite 6 with 5–10 lateral striae on each side.

***Female*:** Pro- and mesotarsi not modified. Abdominal ventrite 6 without lateral striae.

##### Variability.

Beetles from the locality Pap024 are larger (TL-H 3.25–3.65 mm, TL 3.55–4.0 mm, MW 1.75–2.0 mm), with distinctly larger and more robust median lobe and paramere, though of the shape and setation of the median lobe and paramere are the same.

##### Affinities.

The species evidently belongs to the *E. ekari* group due to the discontinuous outline of its median lobe. Based on body size and form, colouration, dorsal surface sculpture, shape of anterolateral hook-like seta of the protarsomere 4, and shape and setation of genitalia, the new species is very similar to *E. soppi*Shaverdo et al., 2012 and, especially, to *E. weylandensis*Shaverdo et al., 2012. However, it differs distinctly from them in presence of the pronotal bead and in that, it is similar to *E. oceai*Shaverdo et al., 2012 and can be as well as distinguished from the co-occurring species, *E. bewaniensis* and *E. oraia* sp. nov.; from *E. oraia* sp. nov. also by not having modified male antennae.

##### Distribution.

Indonesia: Papua Province. This species is known only from the Wano Land (Fig. 15).

##### Habitat.

The specimens were collected from small forest creeks.

##### Etymology.

The species name *ekpliktiki* derives from Greek εκπληκτικός (feminine εκπληκτική) meaning fantastic. The name is an adjective in the nominative singular.

#### Exocelina oraia

Taxon classificationAnimaliaColeopteraDytiscidae

Shaverdo, Surbakti & Balke sp. nov.

3AA5298E-09F7-5371-BFC4-4392C352ED83

http://zoobank.org/6C3D5689-F5EA-4BA3-B1A6-0F28AD993842

Figures 13–14 , 15

Exocelina nr. irianensis #6520: Toussaint et al. 2021: figs 3–6.

##### Type locality.

Indonesia: Papua Province, Puncak Jaya Regency, Puluk area, 03°39'37.0"S, 137°31'14.7"E, 1320 m a.s.l.

##### Type material.

***Holotype*:** male “Indonesia: Papua, Wano Land, Puluk, 1320m, 1.ix.2014, -3.660272 137.5207436, Bennji (Pap020)” (MZB). ***Paratypes***: 14 males, 10 females with the same label as the holotype, one male with an additional label “6520” [green text] (MZB, KSP).

##### Description.

**Figures 13, 14.**
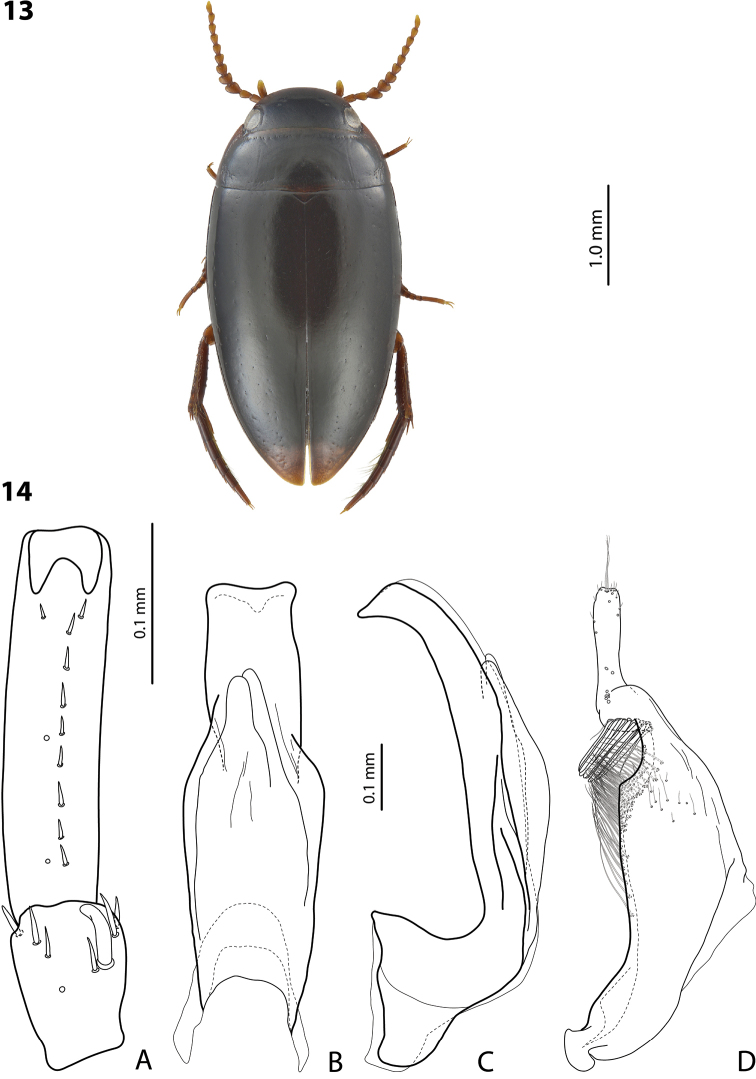
*Exocelina oraia* sp. nov., holotype **13** habitus and colouration **14** male structures **A** right protarsomeres 4 and 5 in ventral view **B** median lobe in ventral view **C** median lobe in lateral view **D** right paramere in external view.

***Body size and form*:** Beetle small to medium-sized: TL-H 3.45–3.85 mm, TL 3.8–4.25 mm, MW 1.85–2.1 mm (holotype: TL-H 3.6 mm, TL 4 mm, MW 1.95 mm), with oblong-oval habitus (Fig. 13).

***Colouration*:** Dorsally piceous (Fig. 13). Head piceous, with slightly paler, dark brown, anterior margin; pronotum piceous, with reddish brown anterior angles and dark brown lateral sides; elytra uniformly piceous; head appendages and proximal part of legs yellowish brown, legs distally brown. Teneral specimens paler.

***Surface sculpture*:** Shiny dorsally, with invisible elytral punctation and weakly impressed microreticulation. Head with relatively sparse punctation (spaces between punctures 1–3 times size of punctures), evidently finer and sparser anteriorly and posteriorly; diameter of punctures smaller than diameter of cells of microreticulation. Pronotum with distinctly finer, sparser, and more evenly distributed punctation than on head, often inconspicuous. Elytra with extremely sparse and fine punctation, often invisible. Pronotum and elytra with weakly impressed microreticulation; head with microreticulation much stronger. Metaventrite and metacoxa distinctly but weakly microreticulate, metacoxal plates with longitudinal strioles and very weak transverse wrinkles. Abdominal ventrites with distinct but weak microreticulation, strioles, and extremely fine and sparse, often invisible punctation, more distinct on abdominal ventrite 6.

***Structures*:** Pronotum without lateral bead, in some specimens (especially characteristic for females) with bead traces or even with narrow bead on lateral sides of pronotum. Base of prosternum and neck of prosternal process with distinct ridge, slightly rounded anteriorly. Blade of prosternal process lanceolate, relatively narrow, slightly convex, with distinct lateral bead and few setae. Abdominal ventrite 6 broadly rounded.

***Male*:** Antenna modified (Fig. 13): antennomeres 3 and 4 strongly enlarged, distinctly larger than other antennomeres, antennomere 5 distinctly enlarged, 6–9 stout. Pro- and mesotarsomeres 1–3 narrow. Protarsomere 4 narrow, with medium-sized, slightly curved anterolateral hook-like seta. Protarsomere 5 ventrally with anterior row of nine and posterior row of three short setae (Fig. 14A). Median lobe with distinctly discontinuous outline; in lateral view, almost straight, with curved downwards, rather narrow, sharply pointed apex; in ventral view, with strong submedian constriction, distal part distinctly narrower than proximal one, apex symmetrical, slightly evenly concave (Fig. 14B, C). Paramere with strong notch on dorsal side and subdistal part short and small; subdistal setae relatively short, thick, flattened; proximal setae more numerous, dense, hair-like, weaker than subdistal ones (Fig. 14D). Abdominal ventrite 6 with 4–8 lateral striae on each side.

***Female*:** Pro- and mesotarsi not modified. Abdominal ventrite 6 without lateral striae. Bead traces or even with narrow bead on lateral margins pronotum present in majority of females.

##### Affinities.

The species evidently belongs to the *E. ekari* group due to the discontinuous outline of its median lobe. The new species is very similar to *E. irianensis*Shaverdo et al., 2012 and *E. wondiwoiensis*Shaverdo et al., 2012 in general appearance, modified male antennae, and structure of the male genitalia, but differs from them in shape of median lobe and setation of the paramere. Additionally, the species shows a stronger tendency to have the lateral bead of pronotum.

##### Distribution.

**Figure 15.**
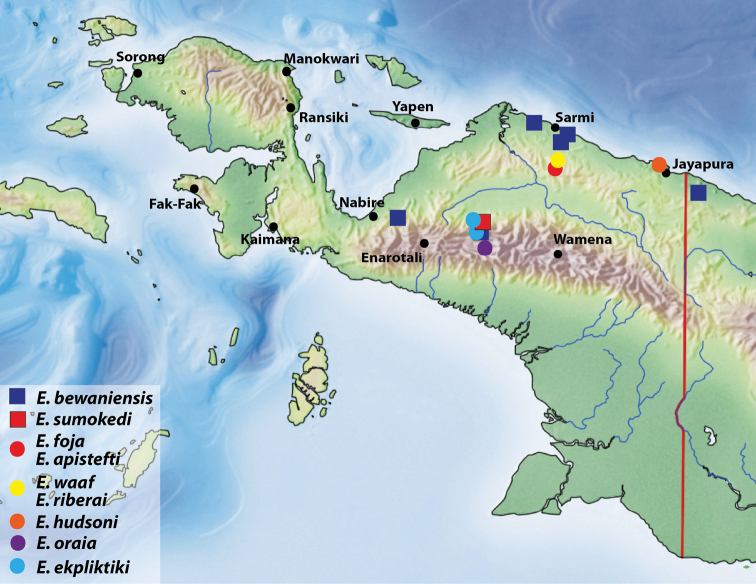
Map of the western part of New Guinea showing the species distribution.

Indonesia: Papua Province. This species is known only from the type locality (Fig. 15).

##### Habitat.

The specimens were collected from small forest creeks.

##### Etymology.

The species name *oraia* derives from Greek *ωραίος* (feminine *ωραία*) meaning nice, lovely. The name is an adjective in the nominative singular.

#### Exocelina bewaniensis

Taxon classificationAnimaliaColeopteraDytiscidae

Shaverdo, Menufandu & Balke, 2014

C08B8B03-EAC4-5142-B285-20F84C78C177

##### New records.

Indonesia: Papua Province: Puncak Jaya Regency (first record): 2 male, 1 female “Indonesia: Papua, Wano Land, creek @ jungle helipad, 870m, 4.ix.2014, -3,584077 137,5042947, Bennji (Pap027)”, one male an additional label “6527” [green text] (KSP). 1 male “Indonesia: Papua, S Iratoi, forest, 168m, 24.v.2015, -3,36070714518427 137,301383111625 (Pap040) Bennji” (KSP).

##### Distribution.

Papua New Guinea: Sandaun Province; Indonesia: Papua Province: Sarmi, Mamberano Raya, Nabire/Paniai, and Puncak Jaya regencies. The present records confirm that this morphologically variable species is broadly distributed in the central-northern part of western New Guinea.

### Corrections

The correct name for *Exocelina athesphatos* Shaverdo et al., 2020 is *Exocelina athesphati* since the species epithet *athesphatos* should be feminine.

#### Exocelina bacchus

Taxon classificationAnimaliaColeopteraDytiscidae

Balke nom. nov.

44537C22-9972-5563-BE22-814381ABB69F

Copelatus (Papuadytes) bacchusi Balke, 1998, not Copelatus bacchusi Wewalka, 1981.

##### Remark.

We provide a replacement name for *Exocelina bacchusi* (Balke, 1998), described as Copelatus (Papuadytes) bacchusi Balke, 1998, since the species name of the latter is preoccupied by Wewalka (1981) and, therefore, it is a junior homonym of *Copelatus bacchusi* Wewalka, 1981. The species stays named for its collector, Mick Bacchus. The name is a noun in apposition.

## Supplementary Material

XML Treatment for Exocelina foja

XML Treatment for Exocelina apistefti

XML Treatment for Exocelina riberai

XML Treatment for Exocelina waaf

XML Treatment for Exocelina hudsoni

XML Treatment for Exocelina ekpliktiki

XML Treatment for Exocelina oraia

XML Treatment for Exocelina bewaniensis

XML Treatment for Exocelina bacchus
